# Supplementary material for: Causal Relationship between Aging and Anorexia Nervosa: A White-Matter-Microstructure-Mediated Mendelian Randomization Analysis
Source: Biomedicines. 2024 Aug 16;12(8):1874. doi: 10.3390/biomedicines12081874 (PMC11351342; doi:10.3390/biomedicines12081874)
Supplement: Supplementary file 1 [file biomedicines-12-01874-s001.zip › Supplementary Figure/Figure S4/Singe SNP plot of MR analysis for TL to ubm-b-1605.pdf]

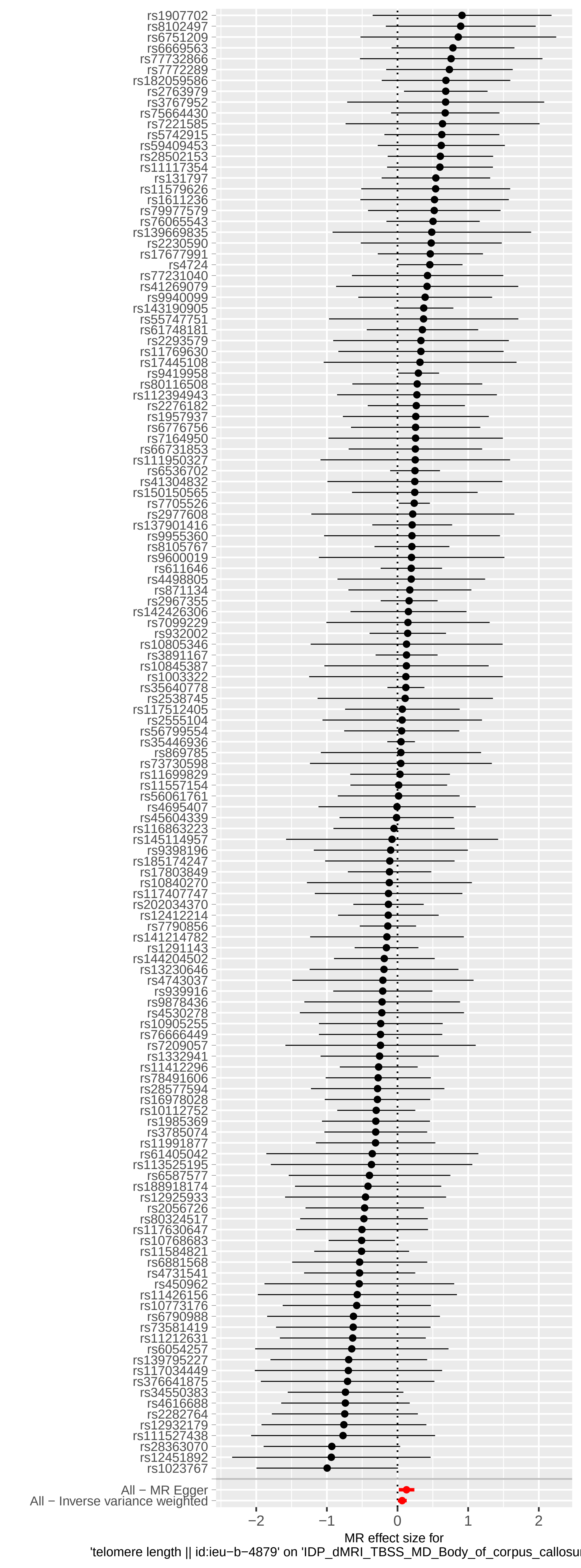

rs1907702
rs8102497
rs6751209
rs6669563
rs77732866
rs7772289
rs182059586
rs2763979
rs3767952
rs75664430
rs7221585
rs5742915
rs59409453
rs28502153
rs11117354
rs131797
rs11579626
rs1611236
rs79977579
rs76065543
rs139669835
rs2230590
rs17677991
rs4724
rs77231040
rs41269079
rs9940099
rs143190905
rs55747751
rs61748181
rs2293579
rs11769630
rs17445108
rs9419958
rs80116508
rs112394943
rs2276182
rs1957937
rs6776756
rs7164950
rs66731853
rs111950327
rs6536702
rs41304832
rs150150565
rs7705526
rs2977608
rs137901416
rs9955360
rs8105767
rs9600019
rs611646
rs4498805
rs871134
rs2967355
rs142426306
rs7099229
rs932002
rs10805346
rs3891167
rs10845387
rs1003322
rs35640778
rs2538745
rs117512405
rs2555104
rs56799554
rs35446936
rs869785
rs73730598
rs11699829
rs11557154
rs56061761
rs4695407
rs45604339
rs116863223
rs145114957
rs9398196
rs185174247
rs17803849
rs10840270
rs117407747
rs202034370
rs12412214
rs7790856
rs141214782
rs1291143
rs144204502
rs13230646
rs4743037
rs939916
rs9878436
rs4530278
rs10905255
rs76666449
rs7209057
rs1332941
rs11412296
rs78491606
rs28577594
rs16978028
rs10112752
rs1985369
rs3785074
rs11991877
rs61405042
rs113525195
rs6587577
rs188918174
rs12925933
rs2056726
rs80324517
rs117630647
rs10768683
rs11584821
rs6881568
rs4731541
rs450962
rs11426156
rs10773176
rs6790988
rs73581419
rs11212631
rs6054257
rs139795227
rs117034449
rs376641875
rs34550383
rs4616688
rs2282764
rs12932179
rs111527438
rs28363070
rs12451892
rs1023767
All − MR Egger
All − Inverse variance weighted
−2
−1
0
1
2
MR effect size for
'telomere length || id:ieu−b−4879' on 'IDP_dMRI_TBSS_MD_Body_of_corpus_callosu
